# Bacteriostatic Antimicrobial Combination: Antagonistic Interaction between Epsilon-Viniferin and Vancomycin against Methicillin-Resistant *Staphylococcus aureus*

**DOI:** 10.1155/2014/461756

**Published:** 2014-03-24

**Authors:** Dayang Fredalina Basri, Lee Wee Xian, Nur Indah Abdul Shukor, Jalifah Latip

**Affiliations:** ^1^School of Diagnostic and Applied Health Sciences, Faculty of Health Sciences, Universiti Kebangsaan Malaysia, Jalan Raja Muda Abdul Aziz, 50300 Kuala Lumpur, Malaysia; ^2^School of Chemical Sciences and Food Technology, Faculty of Sciences and Technology, Universiti Kebangsaan Malaysia, 43600 Bangi, Selangor, Malaysia

## Abstract

Stilbenoids have been considered as an alternative phytotherapeutic treatment against methicillin-resistant *Staphylococcus aureus* (MRSA) infection. The combined effect of **ε**-viniferin and johorenol A with the standard antibiotics, vancomycin and linezolid, was assessed against MRSA ATCC 33591 and HUKM clinical isolate. The minimum inhibitory concentration (MIC) value of the individual tested compounds and the fractional inhibitory concentration index (FICI) value of the combined agents were, respectively, determined using microbroth dilution test and microdilution checkerboard (MDC) method. Only synergistic outcome from checkerboard test will be substantiated for its rate of bacterial killing using time-kill assay. The MIC value of **ε**-viniferin against ATCC 33591 and johorenol A against both strains was 0.05 mg/mL whereas HUKM strain was susceptible to 0.1 mg/mL of **ε**-viniferin. MDC study showed that only combination between **ε**-viniferin and vancomycin was synergistic against ATCC 33591 (FICI 0.25) and HUKM (FICI 0.19). All the other combinations (**ε**-viniferin-linezolid, johorenol A-vancomycin, and johorenol A-linezolid) were either indifferent or additive against both strains. However, despite the FICI value showing synergistic effect for **ε**-viniferin-vancomycin, TKA analysis displayed antagonistic interaction with bacteriostatic action against both strains. As conclusion, **ε**-viniferin can be considered as a bacteriostatic stilbenoid as it antagonized the bactericidal activity of vancomycin. These findings therefore disputed previous report that **ε**-viniferin acted in synergism with vancomycin but revealed that it targets similar site in close proximity to vancomycin's action, possibly at the bacterial membrane protein. Hence, this combination has a huge potential to be further studied and developed as an alternative treatment in combating MRSA in future.

## 1. Introduction

Infection with* Staphylococcus aureus* is a major cause of serious hospital and community-acquired bacteremia worldwide and is associated with significant morbidity and mortality, especially when inappropriately treated [1]. With the emergence of methicillin-resistant* Staphylococcus aureus* (MRSA) in the 1960s, antimicrobial resistance in* S. aureus* has posted a great challenge to clinician treating serious infection, particularly endovascular infection [2]. To date, most of the MRSA strains are resistant to multiple classes of antibiotics and cannot be treated with conventional ß-lactams, making glycopeptides (vancomycin or teicoplanin) the only therapeutic solution [3]. In recent years, emergence of MRSA strains with reduced susceptibility to glycopeptides has emphasized the need for new effective drugs [4]. New antistaphylococcal drugs such as linezolid, which is the first oxazolidinone antibiotic to be approved by the Food and Drug Administration (FDA), have developed and shown efficacy comparable to that of vancomycin for the treatment of pneumonia and soft tissue infections [5, 6]. However, linezolid has not been routinely prescribed because of higher acquisition cost of drug as well as lack of clinical trial and experience compared to vancomycin, thus maintaining vancomycin as a first line of defense in treating MRSA infection [7]. The frequently increasing antibiotic resistance is a serious global problem, and therefore there is a desperate need to explore new class of antibacterial substances, especially from plant-derived sources [8].

Plants are known to produce enormous variety of compounds to protect themselves from being attacked by plant pathogens. In this regard, phytochemicals have been considered as a promising source of novel antibacterial therapeutics due to the successful defense mechanism developed by plants [9]. Stilbenoids are plant secondary metabolites formed in the flavonoid biosynthesis pathway belonging to the phenylpropanoid family [10]. Phenylpropanoids are classified as phytoalexins which are involved in plant responses to various pathogens and herbivores attack [11]. Stilbenoid dimer and trimer used in this study belong to the oligomer stilbenes, which were isolated from* Shorea maxwelliana* and* Shorea johorensis* (Dipterocarpaceae), respectively. Oligomer stilbenes are the main polyphenol compounds known to possess a variety of pharmacological properties such as anti-inflammatory, antibacterial, antiviral, and antifungal [12] activities. Therefore, plant stilbenes have received considerable interest over the past 20 years due to their biological activities and possible pharmacological applications.

In an attempt to overcome the problem of widespread microbial resistance, it is essential to study the new antibacterial agents in combination with the existing antibiotics. In addition to preventing the emergence of resistant mutant strains, combination antimicrobial therapy might be able to increase clinical efficacy as well as to widen the spectrum of antibacterial activity compared to monotherapy [13]. Furthermore, combination therapy can reduce dose regimen of the individual antibiotics and thus undesirable effects of drugs can be reduced [14]. In our last report, it was shown that stilbenoid dimer and trimer displayed anti-MRSA activity with synergistic effect in combination with vancomycin based on microdilution checkerboard (MDC) assay [15]. To our best knowledge, this is the first work on the combined effect of stilbenoids and linezolid using MDC method and using time-kill kinetic study to substantiate the synergistic antimicrobial activity of *ε*-viniferin/vancomycin combination against methicillin-resistant* Staphylococcus aureus* (MRSA).

The present study therefore aimed to confirm the mode of anti-MRSA action of stilbenoids in combination with two selected antibiotics, vancomycin and linezolid, using time-kill assay.

## 2. Materials and Methods

### 2.1. Preparation of Bacterial Inoculum

The bacteria used in this study were methicillin-resistant* Staphylococcus aureus* (MRSA) ATCC 33591 as a reference strain and a clinical HUKM strain. The latter was obtained from Universiti Kebangsaan Malaysia Hospital (HUKM) and was approved by the Research and Ethical Committee of Medical Faculty, UKM. All the bacteria strains were cultured in nutrient agar (NA) and incubated at 37°C for 18 hr. Isolated colonies were inoculated onto slant agar in a bijou bottle for storage at 37°C for 24 hr. The bacteria suspension was adjusted to a turbidity corresponding to a spectrophotometric absorbance at 0.08 at 650 nm, which is equivalent to a bacteria inoculum size of approximately 10^6^ CFU/mL.

### 2.2. Determination of Minimal Inhibitory Concentration (MIC)

The pure compounds used in the study were stilbenoid dimer and trimer, respectively, *ε*-viniferin and johorenol A. The MIC values of *ε*-viniferin and johorenol A against MRSA were determined using twofold serial microdilution method based on [16]. The tested stilbenoids and antibiotics were pipetted in a 96-well plate containing the sterile Mueller-Hinton broth enriched with 2% NaCl before the bacterial suspension was added. The range of final concentration of stilbenoids and linezolid was 0.00078 mg/mL–0.4 mg/mL whereas the range of final concentration of vancomycin was 0.00050 mg/mL–0.2500 mg/mL. A positive control comprised bacteria inoculum in Mueller-Hinton broth whereas the tested compounds in Mueller-Hinton broth were used as negative control. The 96-well plate was then incubated at 37°C for 24 hr. MIC is the lowest concentration of compound and antibiotic showing no turbidity after 24 hr, where the turbidity is interpreted as visible growth of bacteria. Each test was carried out in triplicate.

### 2.3. Determination of Fractional Inhibitory Concentration (FIC)

The combined effect of stilbenoids with antibiotics against MRSA ATCC 33591 and HUKM strains was evaluated from the FICI values for each combination using microdilution checkerboard method. The concentration of individual compound in the combination of stilbenoid and antibiotic which prevented visible bacterial growth was recorded as the MIC of the individual compound in the respective combination. The FICI value was then calculated as follows [17]:

(1) $$\begin{array}{c} \text{FICI}=\text{FIC}\;\text{of}\;\text{stilbenoid}+\text{FIC}\;\text{of}\;\text{antibiotic}, \end{array}$$

where

(2) $$\begin{array}{c} \text{FIC}\;\text{of}\;\text{stilbenoid}=\frac{\text{MIC}\;\text{of}\;\text{stilbenoid}\;\text{in}\;\text{combination}}{\text{MIC}\;\text{of}\;\text{stilbenoid}\;\text{alone}}\\ \text{FIC}\;\text{of}\;\text{antibiotic}=\frac{\text{MIC}\;\text{of}\;\text{antibiotic}\;\text{in}\;\text{combination}}{\text{MIC}\;\text{of}\;\text{antibiotic}\;\text{alone}}. \end{array}$$

Synergistic effect is defined as FICI of ≤0.5; partial synergism as FICI > 0.5 < 1; additivity as FICI = 1; indifference as FICI > 1 ≤ 4; and antagonism as FICI of more than 4.

### 2.4. Time-Kill Study

The rate of bacterial killing over time was performed only on the antibiotic combinations found to be synergistic by microdilution checkerboard method. The time-kill kinetic study of the stilbenoids in combination with antibiotics against both MRSA strains was performed in the microtiter 96-well plates. In each well, the combined agent was added to 0.04 mL Mueller-Hinton broth and 0.05 mL suspension of the bacterial inoculum. The growth control wells comprised only bacteria and 0.05 mL Mueller-Hinton broth. The wells were then incubated at 37°C and viable counts were performed at 0, 2, 4, 6, 8, and 24 hr after addition of treatment agents. At each hour, 0.01 mL of the sample was removed from the wells to be diluted twofold with normal saline (0.9% NaCl). The sample was then spread on Mueller-Hinton agar plates using L-shaped glass rod and incubated for 24 hr at 37°C. Colony count of bacteria of between 30 and 300 CFU/mL for each plate was determined to obtain time-mortality curves by plotting the log_10_ CFU/mL on the* x*-axis and time (hr) on the* y*-axis. Synergistic interaction was defined as *a* ≥ 2 log_10_ decrease in CFU/mL between the combination and the most active agent at 24 hr. Additive or indifference was defined as *a* < 2 log_10_ CFU/mL reduction in colony count at 24 hr by the combination compared with the most active single agent. Antagonism was described as *a* ≥ 2 log_10_ increase in CFU/mL after 24 hr between the combination and the most active agent [18]. The time-kill curves were recorded as a decrease in CFU/mL within a specific time period. Bactericidal and bacteriostatic were, respectively, defined as ≥3 log_10_ or <3 log_10_ *⁡* reduction in colony count at 24 hr compared with the starting inoculum [19].

## 3. Results

The results of anti-MRSA susceptibility test of two stilbenoid compounds (*ε*-viniferin and johorenol A) and linezolid are presented in Table 1. The MIC value of johorenol A against both strains of MRSA and the MIC value of *ε*-viniferin against MRSA ATCC 33591 were 0.05 mg/mL. On the other hand, the MIC value of *ε*-viniferin against HUKM strain was 0.1 mg/mL. As seen in Table 1, linezolid displayed a strong anti-MRSA activity with MIC value of 0.0125 mg/mL against both MRSA strains. On the other hand, Table 2 represented the result of MIC determination of vancomycin which was the lowest among the four antimicrobial agents with MIC value of 0.002 mg/mL against ATCC and HUKM strains.

Table 3 represented the FICI values of the combination studies between the stilbenoids tested and the standard antibiotics. From the microdilution checkerboard assay, only the combination between *ε*-viniferin and vancomycin was synergistic against ATCC 33591 and HUKM strains with FICI values of 0.25 and 0.19, respectively. These FICI values of less than 0.5 indicated a synergistic interaction between *ε*-viniferin and vancomycin. However, combination of *ε*-viniferin and linezolid displayed an indifference reaction against MRSA ATCC 33591 and HUKM strains with FICI values of greater than 1 but less than 4. Johorenol A also showed indifference interaction with vancomycin at FICI values of more than 1 but less than 4 against both strains of MRSA. On the other hand, an additive interaction (FICI = 1) was observed with combined effect of johorenol A and linezolid.

**Figure 1:**
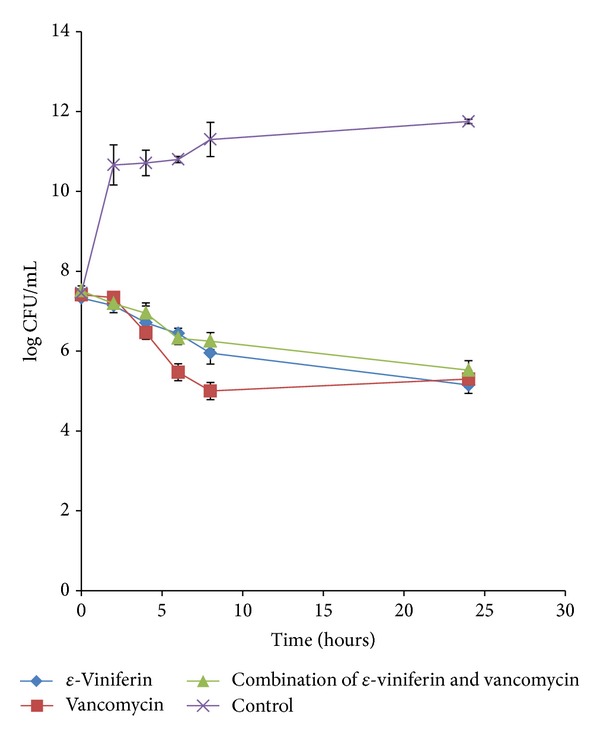
Time-kill growth curves of combination of *ε*-viniferin with vancomycin, *ε*-viniferin alone, and vancomycin alone against MRSA ATCC 33591. The data is presented as a mean of 3 replicates.

Only the combination which displayed synergistic effect will be subjected to time-kill assay (TKA) to confirm such interaction. The combination of *ε*-viniferin and vancomycin which displayed synergistic interaction was disputed through time-kill analysis. From Figure 1, it was clearly shown that bacteriostatic action was observed for *ε*-viniferin singly, vancomycin singly, and *ε*-viniferin-vancomycin combination at the 8th hr against MRSA ATCC 33591 strain. In other words, all treatments exhibited reduction of between 1.3 and 2.4 log_10_ CFU/mL which was then followed by no change in the inhibitory effect against the ATCC strain. TKA curves showed that, at 2 log_10 _ decrease in CFU/mL, *ε*-viniferin alone showed slower rate of inhibition (15.2 hr) compared to vancomycin alone (5.5 hr) but the rate of inhibitory effect in combination treatment was the slowest (24.5 hr). Time-kill assay curve in Figure 1, thereby, indicated that combination treatment showed antagonistic interaction between *ε*-viniferin and vancomycin against MRSA ATCC 33591 strain. This is justified by an increase of more than 2 log_10_ in bacterial counts within a specific time period throughout 24 hr between the combination and the most active agent.

**Figure 2:**
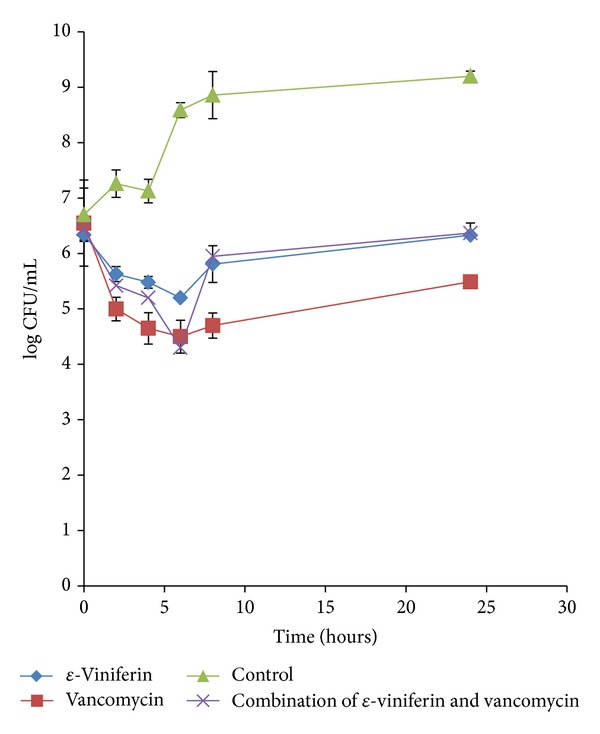
Time-kill growth curves of combination of *ε*-viniferin with vancomycin, *ε*-viniferin alone, and vancomycin alone against MRSA HUKM strain. The data is presented as a mean of 3 replicates.

On the other hand, combination treatment showed a faster inhibitory rate (4 hr) compared to viniferin singly (6 hr) but still slower than vancomycin alone (2 hr) at 1.3 log_10 _ decrease in CFU/mL, against clinical strain as shown in Figure 2. In other words, both combination and vancomycin treatments displayed faster rate of inhibition against HUKM strain compared to *ε*-viniferin singly but all treatments generally showed a reduction of between 1.1 and 2.1 log_10_ CFU/mL indicating bacteriostatic effect. However after the 8th hr, the TKA profile is similar to that against ATCC strain, whereby the combination curve is above that of *ε*-viniferin alone but vancomycin time-kill curve is below that of *ε*-viniferin alone. Time-kill assay curve in Figure 2 indicated that combination treatment showed synergistic effect until the 8th hr after which the interaction between *ε*-viniferin and vancomycin was also antagonistic against the clinical HUKM isolate.

## 4. Discussion

Stilbenoid belongs to a polyphenolic compound which constitutes a large class of resveratrol derivatives such as stilbenoid dimer and trimer, resulting from different oxidative condensation of an individual monomer [20]. Stilbenoid is abundantly distributed in fruits such as grapes, red wine, and vine stems. Numerous research studies have demonstrated many promising biological activities of stilbenoids [21] in which one of those activities includes antibacterial property [22]. The current study demonstrated that *ε*-viniferin and johorenol A showed equal anti-MRSA potency against the reference strain but the clinical strain appeared to demonstrate greater resistance towards *ε*-viniferin than johorenol A. In general, stilbenoid dimer and trimer exert inhibitory effect against both reference and clinical strains of MRSA. This is supported by previous research [23] which showed that there was an antimicrobial activity of trans-*ε*-viniferin isolated from* Vitis amurensis* against* Staphylococcus mutans* and* Staphylococcus sanguis*, establishing stilbenoid dimer as an active antimicrobial agent against most Gram-positive bacteria. Study on the antibacterial properties of methanol extract of chamomile against* S. aureus* found that the phenolic compounds are responsible for the antibacterial activity [24].

Antimicrobial combinations are extensively tested in vitro in the hope of obtaining a synergistic interaction [25], that is, a phenomenon in which one compound enhances the individual activity of another compound in combination and vice versa [26]. Based on the FICI values, *ε*-viniferin reduced the MIC value of vancomycin by eightfold and sixteenfold, respectively, against the reference and clinical strain of MRSA to produce the synergistic effect. The MDC analysis seemed to demonstrate that the interaction with *ε*-viniferin enhanced the activity of vancomycin. Synergy occurs when a combination of two drugs causes inhibition or killing when used at a fourfold lower concentration than that of either component drug used separately [27]. A reduction of more than fourfold in MIC of vancomycin was also observed by *ε*-viniferin against both MRSA strains despite additive interaction [15] based on FICI interpretation by [28]. It is pertinent to emphasize here that if FICI interpretation followed [17] in our previous research, then it may support the present MDC finding. According to [29] when the interaction is synergistic, reduction of the MIC of both antimicrobials occurs, eventually rendering the microorganism susceptible to the level of antimicrobials found in the blood and tissues. The present study demonstrated that there is a synergistic interaction between *ε*-viniferin and vancomycin against MRSA. If this is the case, therefore, it can be assumed that *ε*-viniferin and vancomycin act on the different targets of MRSA based on the understanding that synergism between two antibiotics indicates that their mechanisms of antibacterial action might be different [30].

On the other hand, *ε*-viniferin produced no effect on the antimicrobial activity of linezolid against both MRSA strains as depicted by FIC data. Onefold reduction in the MIC of linezolid indicated that *ε*-viniferin did not interact with linezolid. This is supported by [31] that if the MIC of an antibiotic changed within a onefold dilution in the respective plate, the result was considered as indifference. As such, we can postulate that *ε*-viniferin could possibly have the same mechanism of action as that of linezolid. It has been hypothesized that indifference means that the combined action is the same as with either component [27]. Linezolid is an oxazolidinone antibiotic that acts by inhibiting the initiation complex formation in bacterial protein synthesis [32].

In the current study, the effect of combination of johorenol A significantly reduced the MIC value of vancomycin by more than fourfold, despite exhibiting indifference effect against both MRSA strains. However, when johorenol A is combined with linezolid, the MIC of the latter is significantly reduced by fiftyfold although FIC checkerboard indicated additive effect. This is in agreement with [33] that there was a significant reduction in the MIC values of antibiotics which showed both indifference and additive effects, when used in combination. The present finding on the indifference effect of johorenol A on the bactericidal vancomycin contradicts previous report that the combination of johorenol A with vancomycin displayed synergism against both strains of MRSA [15]. Unfortunately, there is no past literature as far as johorenol A is concerned; therefore, further studies involving time-kill assay are currently ongoing in order to validate these discrepancies. This is because as alternative to the checkerboard method, time-kill assay can be employed to confirm the synergy displayed by the numerical expression of FICI values.

Time-kill test, however, did not support the synergistic outcome of *ε*-viniferin in combination with vancomycin; neither did it represent bactericidal activity of the combined effect. The present data revealed that the combination of *ε*-viniferin and vancomycin produced bacteriostatic action against both MRSA strains as there was less than 3 log_10_ reduction in colony count at 24 hr compared with the starting inoculum. A clear indication of antagonism of the combination can be seen in the time-kill growth curve, whereby the combination growth curve was far above the vancomycin growth curve throughout 24 hr observation (Figure 1) against ATCC strain and during the last 16 hrs of the study duration (Figure 2) against HUKM strain. It has been suggested that antagonism refers to a reduction in the activity of one component in the presence of the other [27]. This explained why the effect of *ε*-viniferin reduced the bactericidal activity of vancomycin. It is important to address that the present TKA study did not demonstrate that vancomycin was bactericidal as a reduction in colony count from the starting inoculum was not ≥3 log_10_ CFU/mL, despite being the most active agent. This is possibly due to microbial regrowth after an initial drop in colony count as frequently observed in time-kill studies. The growth of the resistant subpopulation during exposition to the antimicrobial agent explains the observed regrowth and thus may be interpreted as models of development of resistance [34]. The current study showed that the synergistic result of checkerboard did not correlate with antagonistic outcome of time-kill assay. Therefore, it can be deduced that *ε*-viniferin could well be a bacteriostatic anti-MRSA agent because of a widely established pharmacodynamics concept that antagonism usually occurs when a bacteriostatic agent is combined with a bactericidal agent [35], which in this case is vancomycin. The same finding was observed for the combination of* Quercus infectoria* gall and vancomycin on MRSA 43300 which revealed antagonistic effect by time-kill assay but synergistic effect based on microdilution checkerboard technique. Interestingly, time-kill antagonistic effect was further studied and confirmed by scanning electron microscope and transmission electron microscope analysis [36]. The bactericidal drug more effectively attacks the multiplying bacteria and, hence, if a bacteriostatic drug is used along with it, inhibition of bacterial multiplication may reduce the efficacy of the bactericidal agent [35].

The FICI is the mathematical expression used to measure the inhibitory effect of an interaction [37], whereas the time-kill assay measures the bactericidal activity and killing speed of each combination tested [38]. Time-kill assay is considered as the highest accepted standard for synergy evaluation, despite the fact that checkerboard method has been widely used [39–42]. Thus time-kill antagonistic effect observed in this study was favoured over the synergistic effect displayed by the checkerboard method.

In the present study, two antibiotics were chosen based on their different mechanism of action against MRSA. Vancomycin, antimicrobial agent from glycopeptide class, is a cell wall synthesis inhibitor while linezolid is a protein inhibitor [43]. Antimicrobial agent combinations act on different target site of bacteria that could lead to synergistic effect [44]. Ironically, synergism portrayed by the combination of *ε*-viniferin and vancomycin produced bacteriostatic action whereby the bactericidal activity of vancomycin is reduced in the presence of *ε*-viniferin. It has been reported that benzene ring structure in the phenylpropanoids could exert their biological activities by causing perturbation of the bacterial cell membrane [45]. The present FIC checkerboard method suggested different mechanism of action of *ε*-viniferin from that of vancomycin but similar mechanism of action to linezolid which is in agreement with [46] that phenylpropanoid displayed synergistic interaction with vancomycin suggesting that the interaction of phenylpropanoid is primarily with the bacterial membrane protein. However, the current TKA study was not in accordance with [46] which reported that this interaction enhanced the cell wall inhibitory action of vancomycin, but rather our finding suggested this interaction could adversely affect therapeutic outcomes. The predominant antagonism observed in antimicrobial combination calls for caution in the use of standard antibiotic in combination with phytochemical [47]. Our TKA findings suggested that combination of *ε*-viniferin with vancomycin currently recommended by MDC is not likely to shorten the treatment period due to the lack of synergistic effect. A synergistic combination might have caused rapid and radical bacterial killing, so that the organism gave rise to a smaller number of spores [48]. This is because *ε*-viniferin actually antagonized instead of potentiating the bactericidal action of vancomycin. However, the actual mechanism underlying the interaction of stilbenoids with the present antibiotics is not fully understood and further study would be beneficial to confirm the exact knowledge of mechanism of anti-MRSA action.

## 5. Summary

Generally, it can be concluded that the interaction between *ε*-viniferin and vancomycin results in bacteriostatic action whereby *ε*-viniferin appeared to antagonize the bactericidal activity of vancomycin. In order to prove that resistance of vancomycin is due to the fact that *ε*-viniferin targets the bacterial protein membrane resulting in the molecular changes, future research should address proteomic analysis of MRSA treated with the combined antibiotics in order to elucidate the mechanism of action.

## 6. Recommendation

Further study with regard to morphological and ultrastructural analysis of *ε*-viniferin using scanning electron microscope and transmission electron microscope is underway to confirm the exact mechanism of action and effect of this stilbenoid compound in combination with the standard antibiotics. Hence, this combination has a huge potential to be further studied in view of their mechanism of action and developed as an alternative treatment in combating MRSA in future.

## Figures and Tables

**Table 1:** Determination of MIC values of stilbenoids and linezolid against MRSA ATCC 33591 and HKUM strains.

Concentration (mg/mL)	ATCC 33591			HUKM strain		
	*ε*-Viniferin	Johorenol A	Linezolid	*ε*-Viniferin	Johorenol A	Linezolid
0.4	−	−	−	−	−	−
0.2	−	−	−	−	−	−
0.1	−	−	−	−	−	−
0.05	−	−	−	+	−	−
0.025	+	+	−	+	+	−
0.0125	+	+	−	+	+	−
0.00625	+	+	+	+	+	+
0.00313	+	+	+	+	+	+
0.00156	+	+	+	+	+	+
0.00078	+	+	+	+	+	+

+: presence of bacterial growth.

−: absence of bacterial growth.

Positive control comprises bacterial suspension and Mueller-Hinton broth.

Negative control comprises antimicrobial agent and Mueller-Hinton broth.

**Table 2:** Determination of MIC value of vancomycin against ATCC 33591 and HUKM strains.

Concentration (mg/mL)	Vancomycin	
	ATCC 33591	HUKM strain
0.25000	−	−
0.12500	−	−
0.06250	−	−
0.03125	−	−
0.01560	−	−
0.00780	−	−
0.00390	−	−
0.00200	−	−
0.00100	+	+
0.00050	+	+

+: presence of bacterial growth.

−: absence of bacterial growth.

Positive control comprises bacterial suspension and Mueller-Hinton broth.

Negative control comprises vancomycin and Mueller-Hinton broth.

**Table 3:** Determination of FICI values and interaction effects of stilbenoid and antibiotic combinations against MRSA.

Strains	Agents	MIC (mg/mL)		FIC (mg/mL)		Outcome
		Alone	Combination	FIC	FICI	
ATCC 33591	*ε*-Viniferin	0.05	0.00625	0.125		
	Vancomycin	0.002	0.00025	0.125	0.25	Synergistic
	*ε*-Viniferin	0.05	0.00625	0.125		
	Linezolid	0.0125	0.0125	1	1.12	Indifference
	Johorenol A	0.05	0.05	1		
	Vancomycin	0.002	0.000125	0.062	1.06	Indifference
	Johorenol A	0.05	0.05	1		
	Linezolid	0.0125	0.00025	0.02	1.02	Additive

HUKM strain	*ε*-Viniferin	0.1	0.01250	0.125		
	Vancomycin	0.002	0.000125	0.0625	0.19	Synergistic
	*ε*-Viniferin	0.1	0.00625	0.0625		
	Linezolid	0.0125	0.0125	1	1.06	Indifference
	Johorenol A	0.05	0.05	1		
	Vancomycin	0.002	0.00025	0.125	1.12	Indifference
	Johorenol A	0.05	0.05	1		
	Linezolid	0.0125	0.00025	0.02	1.02	Additive
